# Comment on Montoro-García et al. Beneficial Impact of Pork Dry-Cured Ham Consumption on Blood Pressure and Cardiometabolic Markers in Individuals with Cardiovascular Risk. *Nutrients* 2022, *14*, 298

**DOI:** 10.3390/nu14204266

**Published:** 2022-10-13

**Authors:** Miguel López-Moreno

**Affiliations:** Grupo de Investigación en Biotecnología Alimentaria, Universidad Francisco de Vitoria, 28223 Madrid, Spain; miguel.lopez@ufv.es

Montoro-García et al. [1] recently published in *Nutrients* a randomized controlled trial on the cardioprotective effect of pork dry-cured ham consumption. I would like to point out that the following conclusion regarding ham consumption is misleading:

“This study suggested the beneficial effects of regular dry-cured ham consumption on the improvement of systolic/diastolic blood pressures and facilitated the maintenance of metabolic pathways, which may be beneficial in the primary prevention of cardiovascular disease.”

To make this statement, the effect of dry-cured ham is compared with cooked ham. As Gardner et al. [2] points out, when evaluating the effect of a particular dietary pattern or food on a health outcome, it is vital to establish an appropriate comparator. If the control group consumes a food (cooked ham) with a negative impact on cardiovascular health, it is therefore reasonable that the intervention should have less negative effects [3]. However, we cannot say that this food is heart healthy, especially if we consider studies of isocaloric exchange in which there is evidence of an increased risk of cardiovascular disease associated with the consumption of meat compared to the consumption of plant-based protein [4].

On the other hand, authors should indicate all their interests that may inappropriately influence or bias the results. In this study, two of the authors indicate their affiliation with ElPozo Alimentación S.A., a meat company whose product was used in the dietary intervention. However, in the section on conflicts of interest it is indicated: “All authors declare no conflicts of interests”.
